# *Glycyrrhizae radix* et Rhizoma-Derived Carbon Dots and Their Effect on Menopause Syndrome in Ovariectomized Mice

**DOI:** 10.3390/molecules28041830

**Published:** 2023-02-15

**Authors:** Ying Zhang, Yumin Chen, Xue Bai, Guoliang Cheng, Tianyou Cao, Liyang Dong, Jie Zhao, Yue Zhang, Huihua Qu, Hui Kong, Yan Zhao

**Affiliations:** 1 School of Traditional Chinese Medicine, Beijing University of Chinese Medicine, Beijing 100029, China; 2 School of Chinese Materia Medica, Beijing University of Chinese Medicine, Beijing 100029, China; 3 School of Life Science, Beijing University of Chinese Medicine, Beijing 100029, China; 4 Center of Scientific Experiment, Beijing University of Chinese Medicine, Beijing 100029, China

**Keywords:** carbon dots, *Glycyrrhizae radix* et Rhizoma, menopause syndrome, ovariectomized mice

## Abstract

With the extension of the human life span and the increasing pressure of women’s work and life, menopause syndrome (MPS) refers to a problem that puzzles almost all women worldwide. Hormone replacement treatment (HRT) can effectively mitigate the symptoms but can also exert adverse effects to a certain extent. *Glycyrrhizae radix* et rhizome (GRR) is commonly made into a charcoal processed product, termed GRR Carbonisatas (GRRC), for use in traditional Chinese medicine (TCM). GRRC is widely used to treat MPS and other gynecological diseases. In this study, GRRC was prepared through pyrolysis. Subsequently, GRR-derived carbon dots (GRR-CDs) were purified through dialysis and characterized using transmission electron microscopy, high-resolution transmission electron microscopy, Fourier-transform infrared, ultraviolet, fluorescence, X-ray photoelectron microscopy, and high-performance liquid chromatography. The effects of GRR-CDs on MPS were examined and confirmed using ovariectomized female mice models. The GRR-CDs ranged from 1.0 to 3.0 nm in diameter and with multiple surface chemical groups, as indicated by the results. GRR-CDs can elevate the estradiol (E2) level of healthy female mice. Moreover, GRR-CDs can alleviate MPS using the typical ovariectomized mice model, as confirmed by elevating the estradiol (E2) level and reducing the degree of follicle stimulating hormone (FSH) and luteinizing hormone (LH) and raising the degree of uterine atrophy. The results of this study suggested that GRR-CDs may be a potential clinical candidate for the treatment of MPS, which also provides a possibility for nanodrugs to treat hormonal diseases.

## 1. Introduction

Menopause is defined retrospectively as 12 months of amenorrhea for no other explanations, i.e., the termination of female reproductive capacity; it has been often accompanied by a variety of health issues that may affect all women [1]. Menopausal symptoms that occurred during the perimenopause period containing hot flushes, night sweats, insomnia, vaginal dryness, decreased libido, mood instability and joint pain can be very distressing and may impair wide aspects of women’s lives [2,3,4,5]. Besides, long-term estrogen deficiency may increase the risk of obesity, diabetes, and metabolic syndrome in postmenopausal women [6]. Despite its wide application for treating menopause syndrome (MPS), hormone therapy has numerous side effects (e.g., cardiovascular diseases, risk of thrombosis, and increased incidence of breast cancer [7,8,9]). The research on the treatment of MPS has attracted much interest worldwide.

Carbon dots (CDs) have become a rising star in carbon nanomaterials with a size of smaller than 10 nm in diameter [10]. CDs have aroused wide attention in the medical field for their unique properties (e.g., biocompatibility, excellent water solubility, good conductivity, low cytotoxicity, high electron transfer capability, and environmental friendliness [11,12,13]). CDs have been extensively employed in the biomedical field (e.g., use as probe for in vivo bioimaging [14], electrochemical biosensing [15], cancer diagnosis and therapy [16], and drug/gene delivery [11,17] as well as antibacterial nanomaterials molecules [18]). Our team has studied the therapeutic effects of CDs of many charcoal drugs used in traditional Chinese medicine (TCM) for a few years. Our research has proved that nanomedicines using TCM as carbon source have multiple pharmacological effects and low toxicity [19,20,21]. We wondered if carbon dots with estrogen-increasing effect and low toxicity can be prepared to replace HRT.

*Glycyrrhizae radix* et Rhizoma (GRR) is one of the most frequently used Chinese herbs which is called Gancao in Chinese. It has been documented since the Qin and Han Dynasties. GRR Carbonisatas (GRRC), the product of GRR after high temperature, was first documented in the *Treatise on Febrile and Miscellaneous Diseases* of the Eastern Han Dynasty and was extensively employed in many different diseases thus far (e.g., cardiovascular disease, abdominal pain, MPS, and other gynecological diseases). Our previous research has suggested that CDs generated by high-temperature heating are correlated with the actions of charcoal drugs. The therapeutic effect of GRR-derived carbon dots (GRR-CDs) on MPS was explored according to the TCM clinical application and our preliminary results.

**Figure 1:**
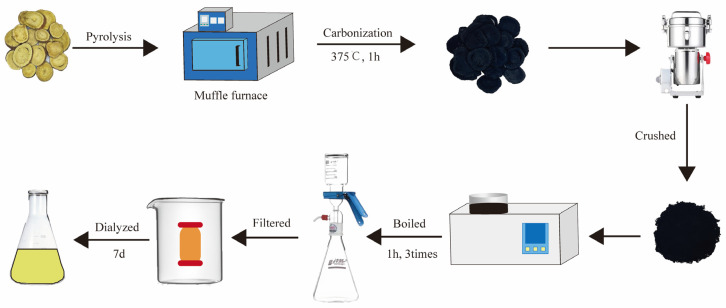
Flow-process chart of the preparation process for *Glycyrrhizae radix* et rhizoma-derived carbon dots (GRR-CDs).

In this study, we performed replicate synthesis three times to ensure that GRR-CDs possessed reproducible properties. Then, GRR-CDs were identified and characterized. The experimental process is shown in Figure 1. First, the result revealed that GRR-CDs is capable of elevating the estradiol level in healthy female mice. Furthermore, the therapeutic effect of GRR-CDs was evaluated in menopausal mice with ovarian removal.

## 2. Results

### 2.1. Characterization of GRR-CDs

The synthesis yield of GRRC was (38.52 ± 0.45)%. The synthesis yield of GRR-CDs was (0.17 ± 0.01)%.

**Figure 2:**
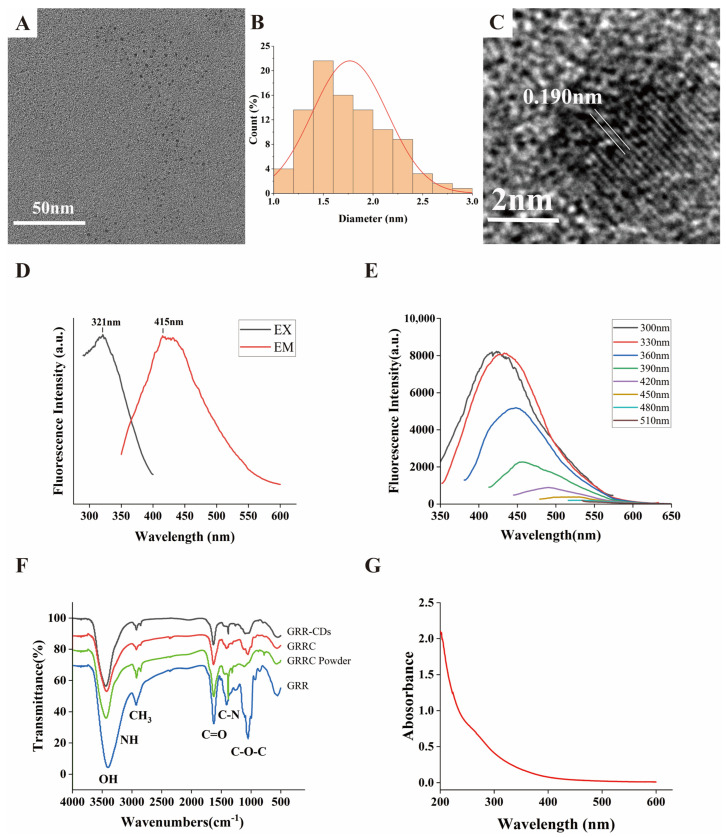
(**A**) Transmission electron microscope (TEM) images of GRR-CDs displaying ultra-small particles. (**B**) Histogram depicting particle size distribution. (**C**) High-resolution TEM (HRTEM) image of GRR-CDs. (**D**) Fluorescence (FL) spectra for excitation and emission. (**E**) Fluorescence spectra of GRR-CDs with different excitation wavelengths. (**F**) Fourier transform infrared spectroscopy spectrophotometer (FTIR) spectrum of GRR-CDs, GRR Carbonisatas (GRRC) without dialysis, GRRC powder before boiling, and Glycyrrhizae radix et rhizoma (GRR). (**G**) Ultraviolet-visible (UV-vis) spectrum of GRR-CDs.

A transmission electron microscope (TEM) was used to describe the morphology and size of GRR-CDs (Figure 2A). Their particle size was 1.77 ± 0.38 nm and distributed between 1.0 and 3.0 nm in diameter (Figure 2B). The high-resolution TEM (HRTEM) image showed that the lattice fringe of GRR-CDs was 0.190 nm (Figure 2C).

The Fluorescence (FL) spectrum showed that the maximum excitation wavelength of GRR-CDs was 321 nm and that the maximum emission wavelength was 415 nm (Figure 2D). The quantum yield (QY) of GRR-CDs was 0.5%. The fluorescence emission spectra of GRR-CDs under different excitations were investigated (Figure 2E). When the wavelength of the excitation light increased from 300 to 510 nm, the emission peaks were all redshifted.

The characteristic peaks of purified GRR-CDs (Figure 2F) were investigated with Fourier transform infrared spectroscopy spectrophotometer (FTIR). The peak at 3433 cm^−1^ was assigned to the absorption bands of O-H and N-H stretching vibrations while C-H stretching vibrations appeared at 2923 cm^−1^. The measurement of 1631 cm^−1^ corresponds to C=O stretching. C-O-C bonds were identified at 1097 cm^−1^, and the peak at 1384 cm^−1^ was attributed to C-N stretching. The FTIR spectrum of GRRC without dialysis, GRRC powder without boiling, and GRR were also described (Figure 2F). The types of main peaks did not show significant difference, while the intensity of absorption peaks changed. The change in the height of the absorption peak indicates that the content of surface groups is different. The reduced groups, such as OH and C-O-C, might be caused by bond breaking during heating.

The ultraviolet-visible (UV-Vis) absorption spectrum (Figure 2G) showed a wide absorption peak between 200 nm and 600 nm, without any evident peak.

### 2.2. X-ray Photoelectron Spectroscopy of GRR-CDs

**Figure 3:**
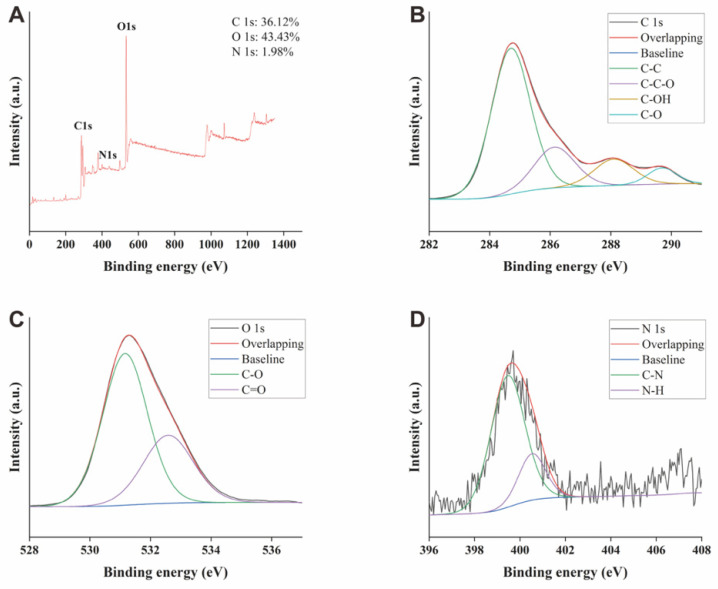
(**A**) Full-scan X-ray spectroscopy (XPS) survey spectra of GRR-CDs. (**B**) C1s, (**C**) O1s, and (**D**) N1s high-resolution X-ray photoelectron spectroscopy spectra.

As depicted in the X-ray photoelectron spectroscopy (XPS) (Figure 3A), the three predominant elements of GRR-CDs comprised C, O, and N elements. To be specific, C and O elements exhibited the highest contents, taking up 36.12% and 43.43% of the total atomic weight, respectively. C1s were divided into four peaks (Figure 3B), corresponding to C-C, C-C-O, C-OH, and C-O groups, respectively; O1s fell into two peaks (Figure 3C), corresponding to C-O and C=O groups, respectively; N1s were separated into two peaks (Figure 3D), corresponding to C-N and N-H groups, respectively. The XPS spectra indicated that there were a variety of functional groups on the surface of GRR-CDs, consistent with the result of FTIR spectrum.

### 2.3. HPLC Profile of GRR and GRR-CDs

**Figure 4:**
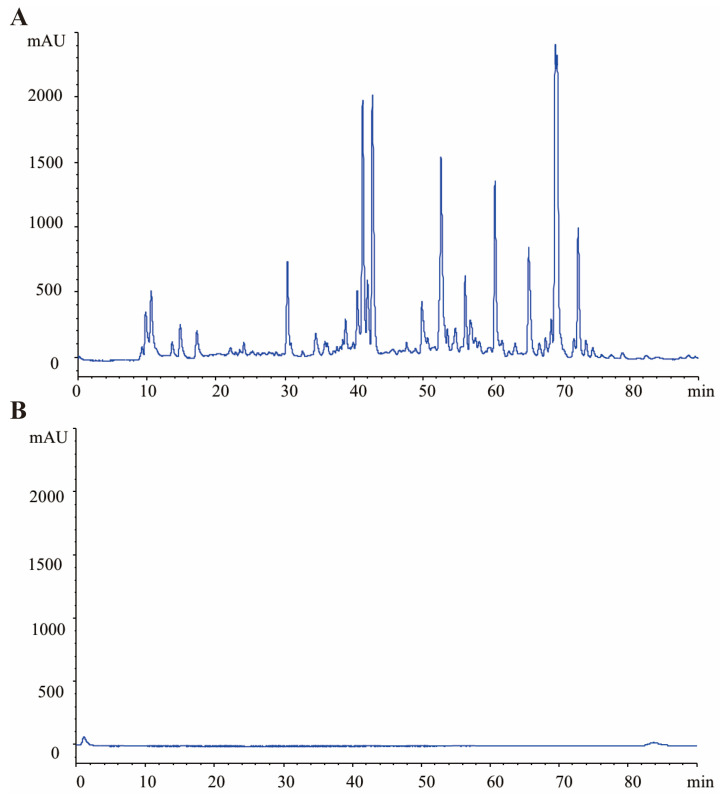
High-performance liquid chromatography (HPLC) profile of (**A**) GRR and (**B**) GRR-CDs.

The High-performance liquid chromatography (HPLC) results of GRR and GRR-CDs were shown in Figure 4. The GRR decoction showed complex compositions and had various small molecule compounds such as lilquiritin apioside, liquiritin, glycyrrhizic acid, and so on. In contrast, GRR-CDs showed no active small molecule compounds under the same condition.

### 2.4. Effect of GRR-CDs on Sex Hormones in Healthy Female Mice

**Figure 5:**
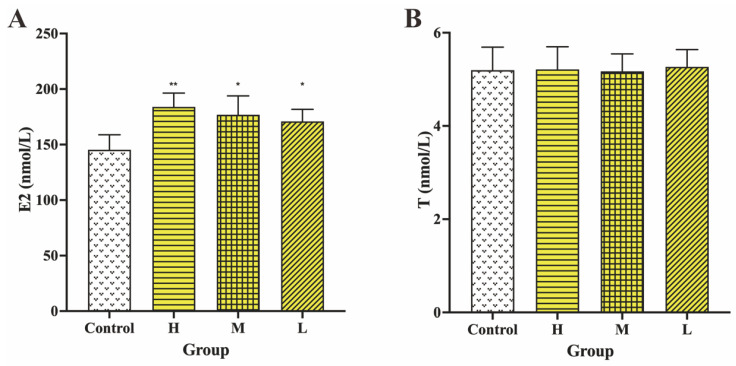
Effects of GRR-CDs on (**A**) estradiol (E2) and (**B**) testosterone (T) in healthy female mice of control, high- (H), medium- (M), and low-dose (L) GRR-CDs groups. * *p* < 0.05 and ** *p* < 0.01 compared vs. control group.

As depicted in Figure 5, estradiol (E2) and testosterone (T) levels in serum were investigated to explore the effect of GRR-CDs on sex hormones in healthy female mice.

Compared with the E2 level in the control group (145.37 ± 13.45 nmol/L), E2 levels significantly increased in the high (184.00 ± 12.45 nmol/L), medium (176.84 ± 17.05 nmol/L), and low (170.90 ± 10.84 nmol/L) doses of GRR-CDs groups.

The T levels of high- (5.21 ± 0.49 nmol/L), medium- (5.10 ± 0.38 nmol/L), and low-dose (5.34 ± 0.38 nmol/L) GRR-CDs groups showed no significant difference (*p* < 0.05) compared with the control group (5.19 ± 0.49 nmol/L).

### 2.5. Effect of GRR-CDs on Sex Hormone in MPS Mice

**Figure 6:**
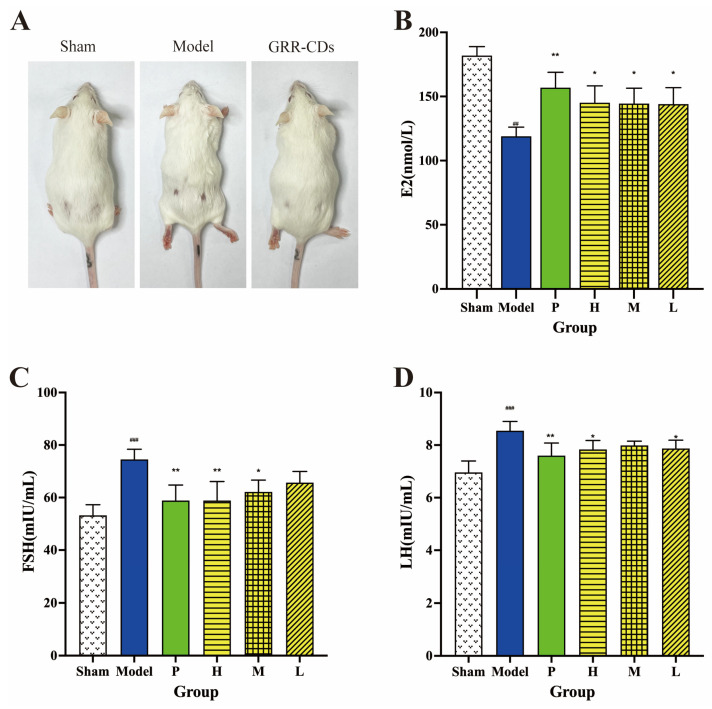
(**A**)Condition of mice hairs in sham operation (Sham), model and GRR-CDs groups after one-week administration. (**B**) Estradiol (E2), (**C**) follicle stimulating hormone (FSH) and (**D**) luteinizing hormone (LH) levels in serum of the sham operation (Sham), MPS model (Model), positive drug (P), high- (H), medium- (M), and low-dose (L) of GRR-CDs groups. ^##^ *p* < 0.01, ^###^ *p* < 0.001 vs. sham operation group, * *p* < 0.05, ** *p* < 0.01 compared vs. model group.

As depicted in Figure 6A, the condition of mice hairs in sham operation, model, and GRR-CDs groups was identified after administration for one week. The hair of mice in the model group was significantly messier than that in the sham operation group. The hair of mice of the GRR-CDs group became smoother than that of the model group.

Sex hormones, (e.g., estradiol (E2), follicle stimulating hormone (FSH), and luteinizing hormone (LH)) were investigated to investigate the therapeutic effect of GRR-CDs on climacteric syndrome.

Figure 6B illustrates the serum E2 level. Compared with the E2 level in the sham operation group (181.88 ± 6.98 nmol/L), E2 levels were significantly down-regulated in the model group (118.96 ± 7.21 nmol/L). E2 levels were significantly up-regulated in the positive (156.84 ± 12.07 nmol/L), high (145.09 ± 13.24 nmol/L), medium (144.47 ± 12.00 nmol/L), and low (144.00 ± 12.89 nmol/L) doses of GRR-CDs groups, as compared with the model group.

Figure 6C presents the serum FSH concentration, detected through ELISA. Compared with sham operation mice (53.22 ± 4.06 mIU/mL), the serum FSH level of model mice (74.51 ± 3.87 mIU/mL) elevated evidently. The mice administrated with estradiol valerate (58.86 ± 5.91 mIU/mL) and high- (58.84 ± 7.25 mIU/mL) and medium- (62.14 ± 4.49 mIU/mL) dose GRR-CDs groups had lower FSH levels. The serum FSH level of the low-dose GRR-CDs group was also lower than the model group, but it was not statistically different.

In addition, as depicted in Figure 6D, the LH level of the MPS model mice (8.55 ± 0.36 mIU/mL) was significantly depressed compared to the sham operation mice (6.96 ± 0.43 mIU/mL). Mice in the positive (7.60 ± 0.48 mIU/mL), high- (7.83 ± 0.34 mIU/mL), and low- (7.87 ± 0.32 mIU/mL) dose GRR-CDs groups had higher levels of serum LH, in sharp contrast to the level in the model group.

### 2.6. Effect of GRR-CDs on Uterus Index

**Figure 7:**
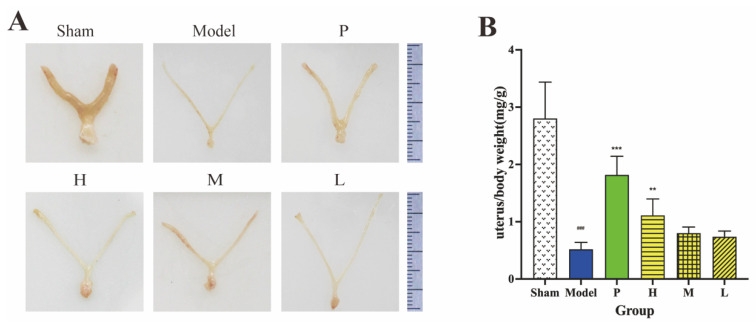
Effect on (**A**) appearance of uterus organ and (**B**) uterus index in sham operation (Sham), MPS model (Model), positive drug (P), high- (H), medium- (M), and low-dose (L) of GRR-CDs. ^###^ *p* < 0.001 vs. sham operation group, ** *p* < 0.01 and *** *p* < 0.001 compared vs. model group.

Figure 7A illustrates the appearance of the uterus organ in six groups. In comparison with the sham group, the uterus shrank; the wet weight of the uterus declined; and the uterus became thinner in the MPS model group after bilateral ovaries were removed for 28 days. The thickness of the uterine wall and uterus wet weight in positive drug, high-, medium- and low-dose groups were increased compared with the result of the model group.

As depicted in Figure 7B, the uterus index of the model group (0.52 ± 0.12 mg/g) plunged to remarkably low levels compared with the sham group (2.80 ± 0.29 mg/g). In contrast to the mice that were only administrated with NS (model group), the above-described levels in the mice administered estradiol valerate (1.82 ± 0.33 mg/g) and high-dose GRR-CDs (1.11 ± 0.29 mg/g) were significantly elevated. The uterus indexes of mice in medium- (0.80 ± 0.11 mg/g) and low-dose (0.74 ± 0.10 mg/g) GRR-CDs groups were also higher than that of the model group, although with no significant difference.

### 2.7. Effect of GRR-CDs on the Histopathological Examination of Uterine Tissue

**Figure 8:**
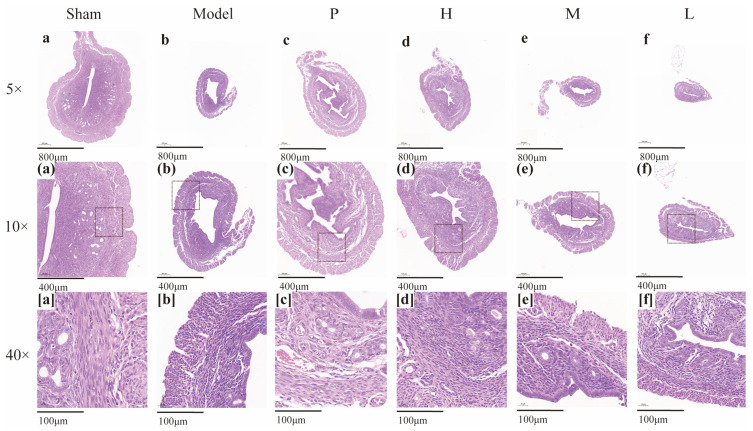
Morphological section of mice uterine tissue of sham operation group (Sham), model group (Model), positive drug group (P), GRR-CDs high- (H), medium- (M), and low-dose (L) group (**a**–**f** Hematoxylin and eosin (H&E) staining; magnification ×5; (**a**)–(**f**) H&E staining; magnification ×10; [**a**]–[**f**] H&E staining; magnification ×40).

Figure 8 presents the histopathological examination of uterine tissue. Compared with the sham operation group, the mouse uterus in the model group showed atrophic lesions, decreased endometrial thickness, and increased necrotic tissue. In addition, the number of glands decreased; the glandular cavity was smaller; and the distribution was uneven. After treatment with the positive drug and different doses of GRR-CDs, the thickness of the endometrium increased; the necrosis smooth muscle tissues were repaired; and the number of glands increased in the positive drug group and high- and medium-dose groups of GRR-CDs. Although the endometrial thickness was increased in the positive drug group, the cells became interstitially edematous and exhibited sparse arrangement.

## 3. Discussion

Estrogen, a sex hormone contained in both men and women, is especially one of the most important hormones in the female body of all ages and has a wide range of biological activities. There are three estrogens naturally produced in the female body: estradiol (E2), estrone (E1), and estriol (E3). E2, the main circulating estrogen, is in the largest quantity of these three estrogens and has the highest affinity for estrogen receptors [22]. These steroids can maintain the basic gender characteristics of women and the development of reproductive organs. Estrogens also play a certain role in the control of energy balance [23]. In addition, they could travel far and interact with multiple organ systems, such as the central nervous system, cardiovascular system, and so on [24]. Psychological stress, excessive exercise, irregular diet, and many other factors in daily life can cause a decline in estrogen. Low levels of estrogen could cause various problems during different periods of women’s lifetime. Experimental evidence has confirmed that estrogen deficiency during the prepubertal period affects craniofacial growth and development [25]. Some researchers considered that the absence of estrogen may cause obesity among menopausal women [26]. Research also supported the idea that the function of the working memory system declines significantly due to decreased estrogen concentrations with advanced aging [27].

MPS has a high prevalence among ethnic groups, and it is correlated with the majority of women worldwide. It is a natural process that occurs in women’s lives when the ovaries have complete (or near-complete) follicular exhaustion, resulting in significantly elevated FSH and very low estradiol levels in serum [28]. It is a retrospective diagnosis usually made after 12 months of amenorrhea [29]. The mechanism of the hypothalamic–pituitary–ovarian axis should be explained to gain more insights into the pathophysiology of menopause. Hypothalamus secretes the gonadotropin-releasing hormone, which regulates the pituitary gland. The pituitary produces gonadotropins, namely FSH and LH, which are responsible for regulating ovarian function. The concentrations of FSH and LH are also regulated by the negative feedback of hormones produced by the ovary, estrogen, and progesterone. FSH is also subjected to a negative-feedback system mediated by the inhibins at the level of the pituitary [30]. The inhibins secreted by the ovarian decrease when the pool of ovarian follicles becomes depleted. Lastly, the depletion of follicles results in estrogen deficiency and the consequent rise in FSH and LH. Thus, the postmenopausal period is characterized hormonally by an elevated FSH and low estradiol levels [31]. Numerous symptoms correlated with menopause will be developed with the disorder of hormone level in women’s bodies, especially with the decline of estrogen level. Vasomotor symptoms have been the most common complaint of women during the menopausal transition (e.g., hot flushes, night sweats, and palpitations [29]). Furthermore, other symptoms (e.g., genitourinary symptoms, sleep disturbance, mood disorder, and cardiovascular risks) are very troublesome and markedly affect the quality of life [32].

Hormone replacement therapy (HRT) by estrogen-only or with progesterone has been confirmed as the most effective way in mitigating menopausal syndrome. HRT is capable of effectively controlling vasomotor symptoms and genitourinary syndrome of menopause [33]. Randomized trials also demonstrate positive effects on reducing bone turnover [34]. Although HRT could effectively reduce symptoms correlated with the decrease in estrogen levels during menopause, the risks of mammalian estrogen application must also be considered. A study by the Women’s Health Initiative (WHI) to evaluate the risks and benefits of hormone therapy in disease prevention clearly demonstrated that the use of estrogen plus progestin in healthy postmenopausal women increased breast cancer, stroke, and heart disease risk [35]. A considerable number of women cannot or choose not to use HRT for its health risks. Accordingly, HRT is usually restricted to moderate or severe symptoms and limited to treat postmenopausal symptoms at minimal dose and duration. More efficacious and better-tolerated alternatives to mitigate menopausal symptoms arouse extensive attention.

CDs become a potential choice for biomedical applications for their characteristics (e.g., cheap and easy synthesis methods, surface functionalization, excellent water solubility, low toxicity, and prominent fluorescence properties) [36]. Our team has investigated CDs that originated from TCM for years and reported similarities between the high-temperature calcination of CDs and the carbonization of traditional Chinese herbs. Our continuous in-depth research has confirmed that CDs lay the material basis for the efficacy of charcoal Chinese herbs, and a set of standardized methods have been finally identified to synthesize and characterize CDs that originate from TCM [37,38,39]. Moreover, our team suggested that CDs with TCM as carbon source had a wide range of bioactivities and with low toxicity. We have verified that CDs that originate from a considerable number of Chinese herbal medicines (e.g., Phellodendri Cortex [40], Cirsium setosum [41], and Cirsii Japonici Herba [42]) exert the notable hemostatic effect. CDs originating from Puerariae lobatae have anti-gout effects [43], and they are capable of increasing the solubility of baicalin in water. Accordingly, CDs derived from TCM with low toxicity have wide and promising applications.

GRR refers to a type of Chinese herbal medicine used commonly in TCM prescriptions for over two thousand years. According to TCM theory, GRR is mainly effective for tonifying fatigue and debilitation, relieving cough and asthma, and reducing drug toxicity. As one of the most frequently used traditional Chinese herbs in *Treatise on Febrile and Miscellaneous Diseases*, it was commonly made into GRRC for use. GRRC has been extensively used to treat gastrointestinal ulcers, abdominal pain, cough, arthritis, and so on. Besides, GRRC also plays a role in the treatment of menopausal palpitation, hot flashes, and other gynecological diseases. From GRR to GRRC, carbon dots were generated in the high-temperature process. Thus, we used GRR as carbon source to exploit a novel nanomedicine with the effects of increasing estrogen.

In this work, GRR-CDs were prepared by a simple and green calcination method at the condition of 375 °C for 1 h. As the only precursor for the synthesis of GRR-CDs, GRR was a type of Chinses medicine with the advantages of low cost, environmental affinity, and high yield, providing the possibility to produce GRR-CDs in a large scale. FTIR and XPS analysis has proved that active groups such as carboxyl and hydroxyl could be found on the surface of GRR-CDs, which may further provide an explanation for the prominent estrogen-increasing bioactivity of GRR-CDs. Compared with the FTIR spectra of GRR, GRRC without dialysis, and GRRC powder before boiling, the change in GRR-CDs surface group content may be the reason for its unique biological effects. HPLC was used to compare the different components between GRR-CDs and GRR, indicating that small molecule substances were removed from the original.

Given that GRR-CDs can significantly elevate estrogen levels in healthy female mice, the effects of GRR-CDs in facilitating pathological estrogen reduction were explored. The complete resection of the bilateral ovaries served as an MPS mice model to simulate the state of MPS in the female body. This method showed several advantages (e.g., high success rate, good stability, and high uniformity). In this study, E2, LH, FSH levels, uterus index, and pathological section of the uterus were selected as the observation targets to explore the effect of GRR-CDs. The positive effects of GRR-CDs in elevating the E2 level has been demonstrated in the MPS model mice. Moreover, the elevated E2 level was indicated by the decreased FSH and LH levels. The above-described conclusion was further verified by the increase in the degree of uterine atrophy. As revealed by this result, GRR-CDs can supplement the E2 in women’s bodies while mitigating the symptoms correlated with MPS. Moreover, this indicates that GRR-CDs may also play a role in the treatment of other diseases related to lower estrogen or low estrogen status in different periods of women’s lives. However, the mechanism of GRR-CDs that elevated the E2 level has not been clarified in this study. The previous research by our team suggested that CDs may affect sex hormone levels by playing a certain role in lipid metabolism. In-depth research should be conducted to determine the underlying mechanisms and broader bioactivities.

## 4. Materials and Methods

### 4.1. Materials

GRR originated from Beijing Qiancao Traditional Chinese Medicine Co., Ltd. (Beijing, China), and GRR-CDs were prepared in our laboratory. Dialysis membranes (cat. No.: HF132640-1m, molecular weight 1000 Daltons, flat width: 45 mm) were purchased from Beijing Ruida Henghui Technology Development Co., Ltd. (Beijing, China). Benzylpenicillin potassium for injection was offered by Jiangxi Keda Animal Pharmaceutical Co., Ltd. (Fuzhou, China). Mouse E2, T, FSH, and LH Elisa kits were offered by Kete Biotechnology Co., Ltd. (Yancheng, China). All other commercial chemicals and reagents employed in this study were purchased from Sino-pharm Chemical Reagents Beijing (Beijing, China) and were of analytical reagent grade. All experiments were performed with deionized water.

### 4.2. Animals

The protocols of this study conformed to the Guide for the Care and Use of Laboratory Animals and gained approval from the Committee of Ethics of Animal Experimentation of the Beijing University of Traditional Chinese Medicine (with the ethic code of BUCM-2022032401-1050). Female adult Kunming mice (weighing 25.0 ± 2.0 g) were provided by the SiBeiFu (Beijing, China) Biotechnology Co., Ltd. All animals were placed in a controlled environment with the ambient temperature of 24.0 ± 1.0 °C, relative humidity of 55–65%, under a 12 h light/dark cycle, and with free access to food and water based on adaptive feeding for 7 days.

### 4.3. Preparation of GRR-CDs

GRR was placed in crucibles and covered with aluminum foil to form a sealed place. Subsequently, the GRR was calcined at 375 °C for 1 h in a pre-heated muffle furnace (TL0612 muffle furnace; Beijing ZhongKe Aobo Technology Co., Ltd.; Beijing, China). GRRC after heating was weighed to determine the synthesis yield (SY) as follows:

SY(GRRC) = GRRC weight (g)/GRR weight before carbonization(g) × 100%(1)

The GRRC was crushed into a fine powder after the furnace cooled to ambient temperature. GRR-CDs were purified by using the dialysis method. A total of 50 g powder was removed, mixed with 1500 mL deionized water (DW), and boiled at 100 °C three times in a water bath for one hour each time. The residue was removed by filtration through a 0.22 μm filter membrane, and the obtained solution was dialyzed against DW for 7 days using a dialysis membrane with a molecular weight cut-off of 1000 Da to obtain the purified GRR-CDs and then stored at 4 °C for further characterization and use. The GRR solution was prepared by boiling at the same condition and without dialysis. The weight of GRR-CDs was weighed after lyophilization. The SY of GRR-CDs was calculated as follows:

SY(GRR-CDs) = GRR-CDs weight (g)/GRR weight before carbonization(g) × 100%(2)

### 4.4. Characterization of GRR-CDs

The morphology and size of GRR-CDs were characterized using a transmission electron microscope (TEM, JEM-2100 electron, Japan Electron Optical Laboratory, Tokyo, Japan) with an acceleration voltage of 200 kV. The atomic lattice fringes of the GRR-CDs were examined using high-resolution TEM (HRTEM, JEN1230, Japan Electron Optical Laboratory, Tokyo, Japan). GRR-CDs were performed in KBr pellet, and the organic functional groups were recorded on a Fourier transform infrared spectroscopy spectrophotometer (FTIR, Thermo Fisher Scientific, FremontCA, USA). The UV-Vis spectrum and fluorescence properties were acquired using UV-Vis (CECIL, Cambridge, UK) and fluorescence spectroscopy (F-4500, Hitachi, Tokyo, Japan), respectively. The surface chemical groups and elemental composition information were analyzed by X-ray photoelectron spectroscopy (XPS, ESCALAB 250Xi, Thermo Fisher Scientific, Fremont, California, USA). 

### 4.5. Fluorescence Quantum Yield of GRR-CDs

A quantity of 0.1 M quinine sulphate (quantum yield, 54%) served as a standard solution to determine the FQY of GRR-CDs, and was calculated as follows:

(3) $$Q_{\mathrm{CDs}}=Q_{R}\;\times \;\frac{I_{\mathrm{CDs}}}{I_{R}}\times \frac{A_{R}}{A_{\mathrm{CDs}}}\times \frac{\eta_{\mathrm{CDs}}^{2}}{\eta_{R}^{2}}$$

where *Q* represents the QY; *I* denotes the integral area of emission intensity; “*A*” expresses the absorbance at 321 nm; “η” is the refractive index of solvent. The subscripts CDs and R are GRR-CDs and standard, respectively. *A_R_* and *A_CDs_* were kept below 0.05 to minimize the effect of reabsorption.

### 4.6. GRR-CDs Fingerprint Analysis

High-performance liquid chromatography (HPLC) was used to evaluate the individual components before and after pyrolysis and dialysis to compare the difference between GRR and GRR-CDs. The fingerprint analysis was conducted using a ZORBAX-C18 column (250 × 4.6 × 5 mm, Orochem Technologies, Illinois, USA) based on an Agilent series 1260 HPLC instrument (Agilent Technologies, Waldbronn, Germany) equipped with a quaternary pump, a diode-array detector, a degasser, an autosampler, as well as a column compartment. The methanol extract of GRR and GRR-CDs was prepared and then processed under the same detection conditions. The mobile phase A (acetonitrile) and B (4% phosphoric acid) were filtered through 0.22 µm cellulose acetate membrane filters (JinTeng, Tianjin, China) prior to use. The gradient program is elucidated in the following: 0–15 min, 10–20% A and 90–80% B; 15–30 min, 20% A and 80% B; 30–40 min, 20–30% A and 80–70% B; 40–50 min, 30–10% A and 70–90% B. Furthermore, the column was held at 35 °C; the flow rate was set to 1.0 mL/min; and the detection wavelength was 254 nm.

### 4.7. Treatment on Healthy Female Mice

Female KM mice (*n* = 24) were randomly assigned to four groups of six animals each as follows: control, high- (H), medium- (M), and low-dose (L) GRR-CDs treatment groups. Mice in the control group were administrated with NS (normal saline). The high- (H), medium- (M), and low-dose (L) GRR-CDs treatment groups were administered 1.6 mg/kg, 0.8 mg/kg, and 0.4 mg/kg of GRR-CDs, respectively. Drugs were given by intragastric administration once a day continuously for 14 days.

### 4.8. MPS Mouse Model and Drug Treatment

Female KM mice (*n* = 36) were randomly assigned to six groups of six animals each as follows: sham operation (Sham), MPS model (Model), positive drug (P), and high- (H), medium- (M), and low-dose (L) GRR-CDs treatment groups. Bilateral ovaries of mice were removed to build the MPS model except for the sham operation group. Mice that only removed some adipose tissue by the side of the ovary were employed as the sham group. Then the mice were applied the penicillin to the suture after the surgery and identified for 5 days. The sham operation and MPS model groups were treated by NS, and the positive drug group was administrated with estradiol valerate tablets (0.15 mg/kg, BAYER, Leverkusen, German). The high- (H), medium- (M), and low-dose (L) GRR-CDs treatment groups were administered 1.6 mg/kg, 0.8 mg/kg, and 0.4 mg/kg of GRR-CDs, respectively. Drugs were given by gavage administration once a day, and the treatment duration was 28 days.

### 4.9. Effect of GRR-CDs on the Uterus Index in MPS Mice

The body weight of mice and the wet weight of the uterus were weighed and then recorded before and after blood collecting, respectively, to determine the uterus index (UI) as follows:

UI = uterus wet weight (mg)/body weight (g)(4)

### 4.10. Detection of Sex Hormones in Serum

After the treatment, blood samples were acquired by removing the eyeballs of mice. Next, the samples were centrifugated at 3000 rpm for 10 min to obtain the serum. E2 and T levels in healthy female mice and E2, FSH, and LH levels in the MPS mice were examined using Elisa kits (Kete Biotechnology Co., Ltd., Yancheng, China) in accordance with the manufacturer’s instructions, respectively.

### 4.11. Histopathological Examination of Uterus Tissue

The right side of uterus tissue samples were fixed in 10% neutral-buffered formalin at 4 °C for more than 48 h, dehydrated, embedded in paraffin, sectioned, and then stained with hematoxylin and eosin (H&E) for histopathological examination. Morphological changes were compared among the sham operation, model, positive drug, and GRR-CDs-treated groups.

### 4.12. Statistical Analysis

All data analyses were performed using IBM SPSS statistics software (version 26). Results were shown as means ± standard deviation (SD). Differences between groups were performed by one-way ANOVA followed by the least significant difference test. *p* value < 0.05 indicated statistical differences and *p* value < 0.01 indicated statistically significant differences.

## 5. Conclusions

In this study, we successfully synthesized GRR into GRR-CDs by one-step pyrolysis. To the best of our knowledge, for the first time, this study proves that GRR-CDs could elevate the E2 level, decrease FSH and LH levels in serum, and moreover reduce the degree of uterine atrophy. This not only suggests the potential of GRR-CDs as a drug for alleviating menopause syndrome and its related symptoms, but also provides a potential possibility for nanodrugs to treat hormonal diseases.

## Data Availability

The data supporting this study’s findings are available from the corresponding author upon reasonable request.

## Sample Availability

Samples of the compounds of GRR-CDs are available from the authors.
